# The 21st annual Bioinformatics Open Source Conference (BOSC 2020, part of BCC2020)

**DOI:** 10.12688/f1000research.26498.1

**Published:** 2020-09-21

**Authors:** Nomi L. Harris, Peter J.A. Cock, Christopher J. Fields, Karsten Hokamp, Jessica Maia, Monica Munoz-Torres, Morgan Taschuk, Yo Yehudi

**Affiliations:** 1 Lawrence Berkeley National Laboratory, Berkeley, CA, 94720, USA; 2 Information and Computational Sciences, James Hutton Institute, Dundee, DD2 5DA, UK; 3 Carver Biotechnology Center, University of Illinois at Urbana-Champaign, Urbana, IL, 61801, USA; 4 Smurfit Institute of Genetics, Trinity College Dublin, Dublin, D02 PN40, Ireland; 5 BD Technologies, Durham, NC, 22709, USA; 6 Environmental and Molecular Toxicology, Oregon State University, Corvallis, OR, 97331, USA; 7 Ontario Institute for Cancer Research, Toronto, Ontario, M5G 0A3, Canada; 8 Wellcome Trust, London, NW1 2BE, UK

**Keywords:** bioinformatics, open source, open science, online conference

## Abstract

Launched in 2000 and held every year since, the Bioinformatics Open Source Conference (BOSC) is a volunteer-run meeting coordinated by the Open Bioinformatics Foundation (OBF) that covers open source software development and open science in bioinformatics. Most years, BOSC has been part of the Intelligent Systems for Molecular Biology (ISMB) conference, but in 2018, and again in 2020, BOSC partnered with the Galaxy Community Conference (GCC). This year’s combined BOSC + GCC conference was called the Bioinformatics Community Conference (BCC2020, bcc2020.github.io).

Originally slated to take place in Toronto, Canada, BCC2020 was moved online due to COVID-19. The meeting started with a wide array of training sessions; continued with a main program of keynote presentations, talks, posters, Birds of a Feather, and more; and ended with four days of collaboration (CoFest). Efforts to make the meeting accessible and inclusive included very low registration fees, talks presented twice a day, and closed captioning for all videos. More than 800 people from 61 countries registered for at least one part of the meeting, which was held mostly in the Remo.co video-conferencing platform.

## Introduction

BOSC 2020, the 21st annual Bioinformatics Open Source Conference, took place online in July 2020 in conjunction with the Galaxy Community Conference (GCC). This joint event, the Bioinformatics Community Conference ( BCC2020), was the second time BOSC and GCC joined forces; the first was GCCBOSC 2018 in Portland, Oregon, USA.

BCC2020, which was held mostly in the Remo.co video-conferencing platform, started with a wide array of training sessions; continued with a main program of keynote presentations, talks, posters, Birds of a Feather, and more; and ended with four days of collaboration (CoFest). Over 800 people from 61 countries registered for at least one part of the meeting, with over 500 registered for the main conference. (In comparison, total attendance at GCCBOSC 2018 was just over 300, with 243 attending the main meeting.) Of attendees who responded to the post-BCC2020 survey, over two thirds indicated that this was their first time attending BOSC or GCC.

## Going virtual

BCC2020 was originally going to be held in Toronto, Canada, but due to the COVID-19 pandemic the organizers decided to hold the conference online. This represented a challenge but also an opportunity, as many people from all over the world participated, attracted in part by the low registration fees and timezone-friendly programming (with all sessions held in the western hemisphere and then repeated for the eastern hemisphere).

Most of the people on the BCC2020 organizing committee were experienced at running in-person meetings, but none of us had ever organized a big online conference before, so we had to figure out a lot as we went along. You can read more here about the decisions we made, the challenges we encountered, and the advantages of the specific technologies we chose.

As we planned our online meeting, we realized that we needed to find technology solutions that would:

- convey scientific content through keynotes, talks and posters in an online format
- enable interactive sessions for training
- allow participants to ask questions of presenters, and presenters to respond
- capture some of the social aspects of a conference
- support active, persistent communication channels for all participants
- promote accessibility and inclusion

We chose Discord (which is open source) for text chat and discussion. Discord allows asynchronous communication in multiple topical channels and also permits integration of ‘bots’ to automatically post updates (for example, listing the talks in the next session). For sharing information about the conference, we used the BCC2020 website ( http://bcc2020.github.io/), Sched ( https://bcc2020.sched.com/), and an “Info-Hub” Google doc that could be updated on the fly.

**Figure 1.**
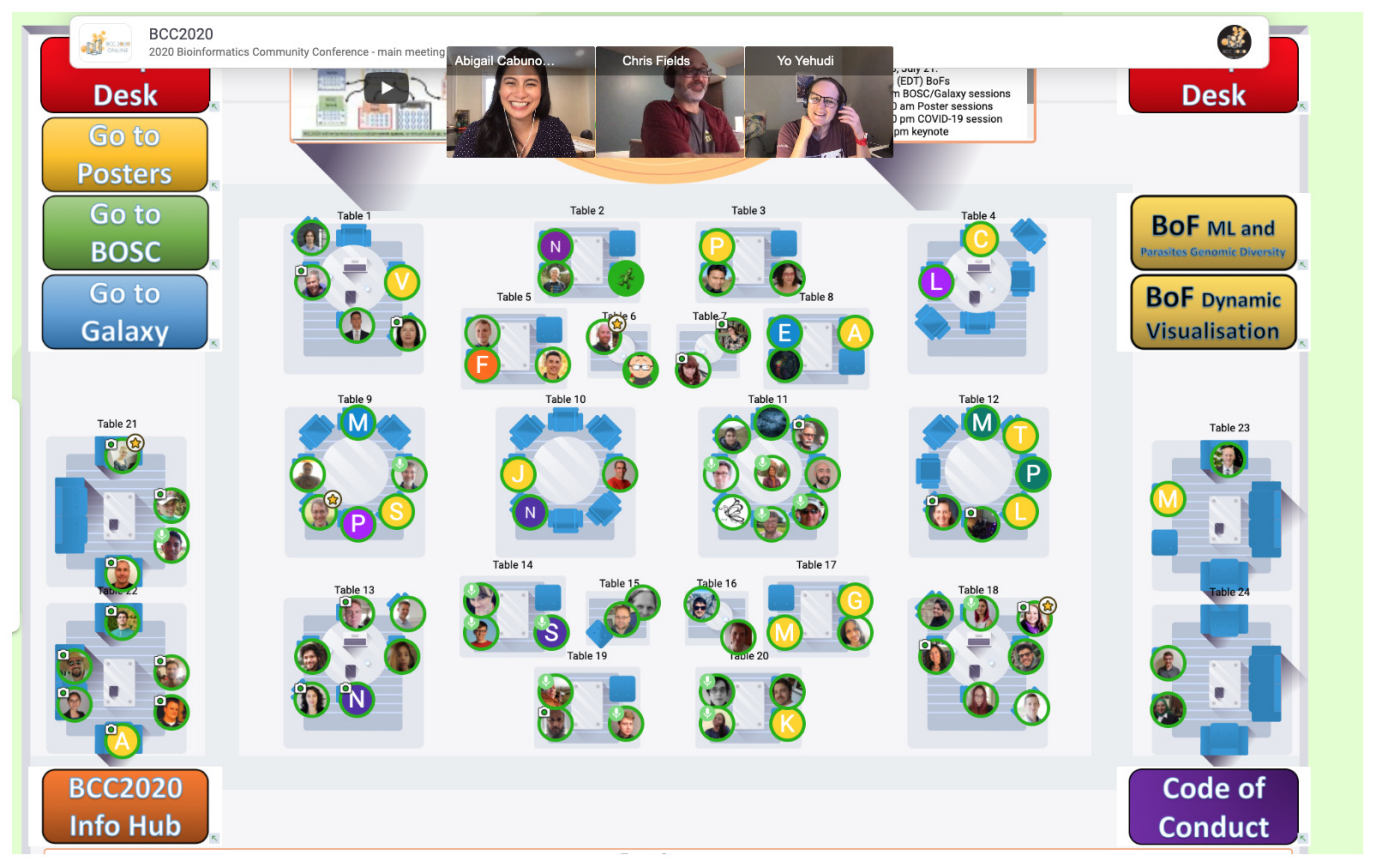
Example of “Table View” in Remo. Each table represents a separate virtual space in which participants who occupy one of the seats can chat with each other. Participant videos are shown as thumbnails (as shown) or tiled across the whole screen like in a Zoom “gallery”. The coloured, clickable buttons on the left and right sides are connections (or bridges) to other Remo event spaces (such as parallel sessions) or to online resources, such as the Code of Conduct or the conference info hub.

**Figure 2.**
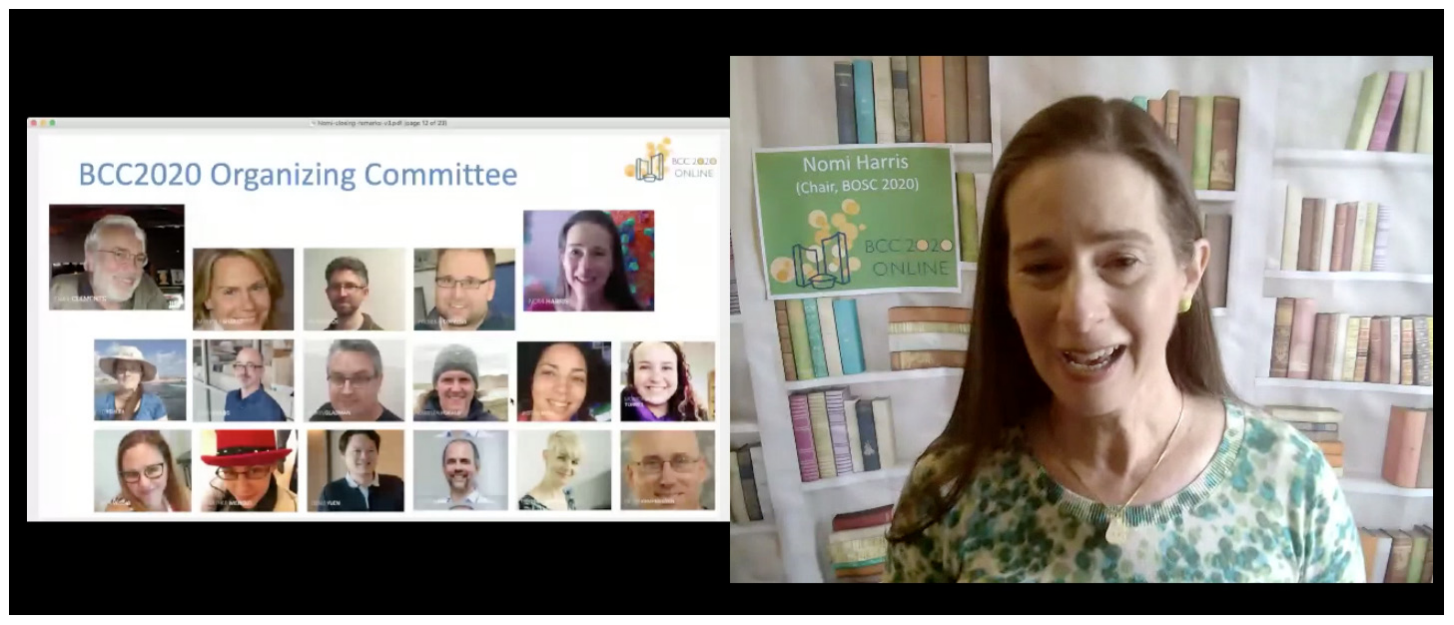
Example of “Presentation View” in Remo. In this mode, which is like a webinar, up to six presenters can share their webcam and screen with the audience.

For the talks and training sessions, we decided to use a new videoconferencing platform, Remo.co, which represents online meetings as virtual buildings with tables at which attendees can mingle. This “table mode” ( Figure 1) felt similar to wandering the conference hall at an in-person event, encountering and chatting with people you know and meeting new ones. For talks, Remo’s “presentation mode” ( Figure 2) offered a webinar-like interface. Keynote talks were delivered live in their home hemisphere; other talks were pre-recorded by the presenters, uploaded by us to Vimeo, and streamed in Remo during the sessions in both hemispheres.

**Figure 3.**
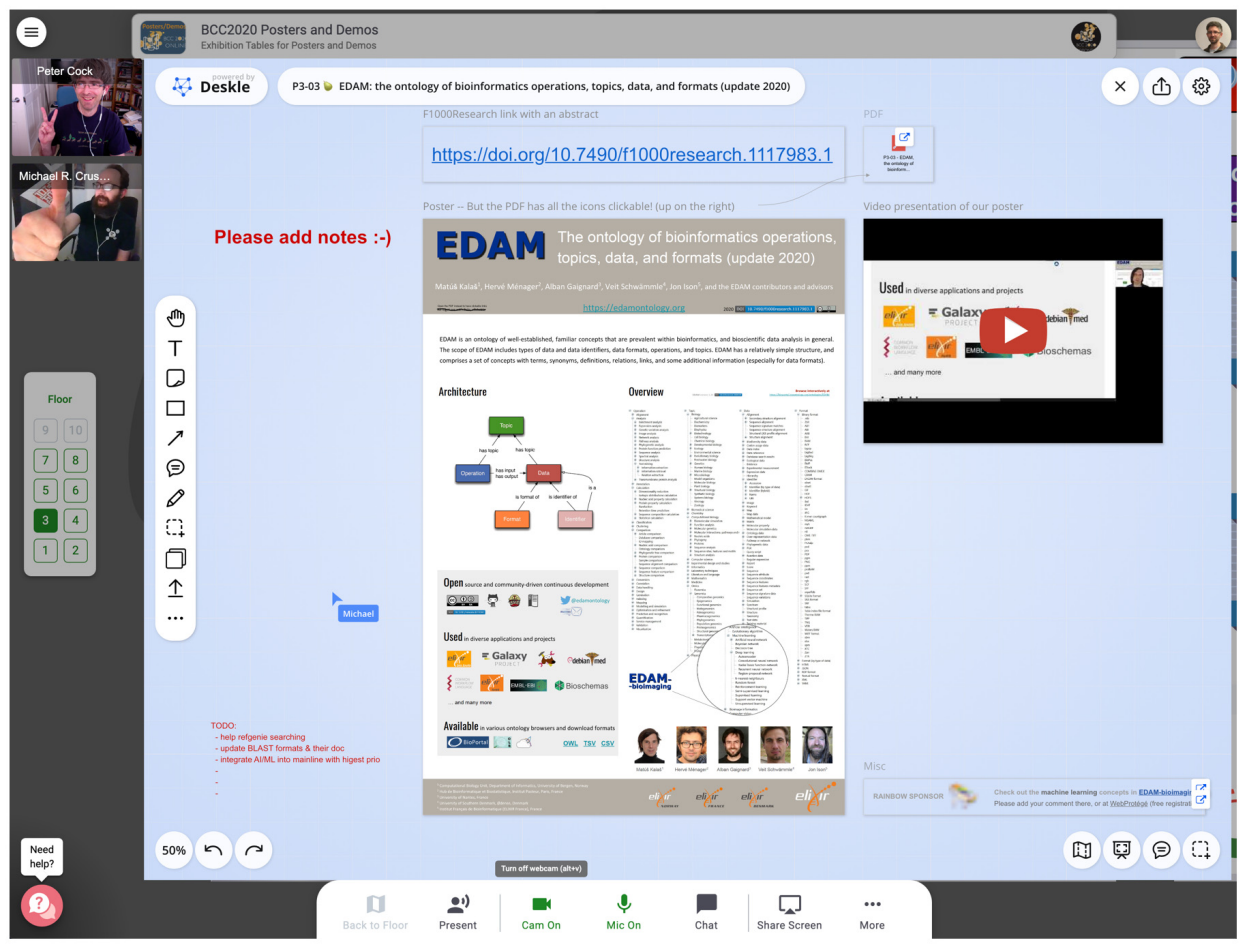
Example of a poster “whiteboard”. Posters at BCC2020 used the Remo whiteboard feature, which allowed poster presenters to attach multimedia including PDFs, videos, and links. The two small pictures on the left are the webcams of people who were looking at that poster and who chose to enable their video.

Remo offered an easy way for participants to jump between parallel tracks (represented as a “BOSC building” and a “Galaxy building”). We also had a “poster building”, which was one of the best features of our online setup. Each poster presenter was assigned a “table” with a digital whiteboard on which they could display material including PDFs, videos, links, etc., and they could chat (via text, audio, or video) with participants who visited their poster ( Figure 3).

## Accessibility and inclusion

The organizers of BCC2020 endeavored to make the conference as inclusive as possible. The online format helped with some aspects of this goal. Registration prices were a fraction of what they would have been for an in-person event, and were further discounted for students and for attendees from low and middle income countries. Since even the very low fees were a barrier for some would-be attendees, both BOSC (via the OBF) and Galaxy awarded registration waivers to selected applicants. Perhaps most significantly, although there can be costs associated with attending an online meeting, such as childcare and internet service, there were no travel expenses. This enabled many people who live far from the usual conference locations (mostly Europe and North America) to attend BOSC or Galaxy for the first time.

To make the conference accessible to people around the globe, BCC2020 events were held twice a day: once in their original Toronto time zone (North American Eastern Time), and again 12 hours later for those in the Eastern hemisphere. The Toronto times were in the middle of the night for some international attendees, who were very grateful to be able to participate in the conference at more reasonable hours. To allow even more flexibility, the video recordings of talks were made available to attendees to watch whenever they chose. This was helpful to attendees who had family demands or who wanted to watch talks that were scheduled simultaneously in the BOSC and Galaxy tracks.

Thanks to generous sponsorship from eLife, we added closed captioning (CC) to both prerecorded and live talks. Over 25% of attendees (as reported in the post-conference survey) utilized these, and many--including those who did not have hearing issues--shared their appreciation. “Fantastic, very helpful,” one respondent commented about the CC. “I have good hearing, but it helped me digest more effectively anyway. (I also turn on CC on Netflix for the same reason!)” Another noted, “It was very useful. My audio was not working for a few moments, and the CC allow[ed] me to still follow the conference.” The appreciation and high uptake we saw with the CC is an example of how increasing accessibility can improve the conference experience for a wide range of participants.

**Figure 4.**
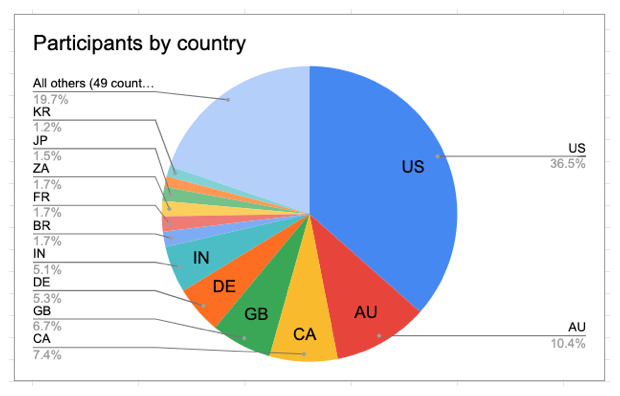
BCC2020 participants by country (based on the country they provided in their address during registration).

All of these factors--low registration fees, no travel required, registration fellowships, talks available at convenient times for both hemispheres as well as asynchronously, and closed captioning--contributed to the large, diverse attendance at BCC2020. Participants hailed from 61 countries ( Figure 4), many of which had never been represented before at BOSC. Australia had an unusually large turnout (beating Canada, Great Britain, and Germany), probably due in part to the recruitment efforts of the organizers from Galaxy Australia. In total, 32% of participants were from outside of Europe and North America, including 47 people from Africa and 25 from South America.

Nevertheless, despite our best efforts to promote diversity and inclusion, we did not see proportional attendance from people belonging to underrepresented groups; the demographic of the meeting was still mainly white and mainly from high economic status countries. Out of the 91 attendees who responded to the survey question, “Do you see yourself as a member of a group that is typically underrepresented at bioinformatics conferences?” only 4 indicated that they were of African or Indigenous heritage, with an additional 9% saying they were Latinx. Only 6 people said they were over 50 years old. Encouragingly, though, 35% of the people who answered that question said they were women, which is impressively high for a bioinformatics conference. (Note, however, that the number of responses was too small to draw any sweeping conclusions.) The organizers will continue to look for ways to promote future conferences to a wider audience and encourage more diverse participation.

## Conference program

### Training sessions

Like GCCBOSC 2018, BCC2020 started with optional training sessions. 60 training sessions covering a wide range of topics were offered, some in one hemisphere and some in both. 473 people (over half of the people who registered for BCC2020) signed up for one or more training sessions; some attended as many as 10. Some training workshops focused on specific tools or analysis ecosystems (e.g., Reactome, Nextflow, Terra, and of course Galaxy); some discussed a field such as machine learning or RNA-Seq analysis; some introduced useful general-purpose tools such as Git and Docker; and some were data-focused. A few workshops focused more on outreach than on coding, with topics such as "Building communities with open source + open science." The full list of BCC2020 training sessions is available at https://bcc2020.sched.com/overview/subject/Training.

### Keynotes

**Figure 5.**
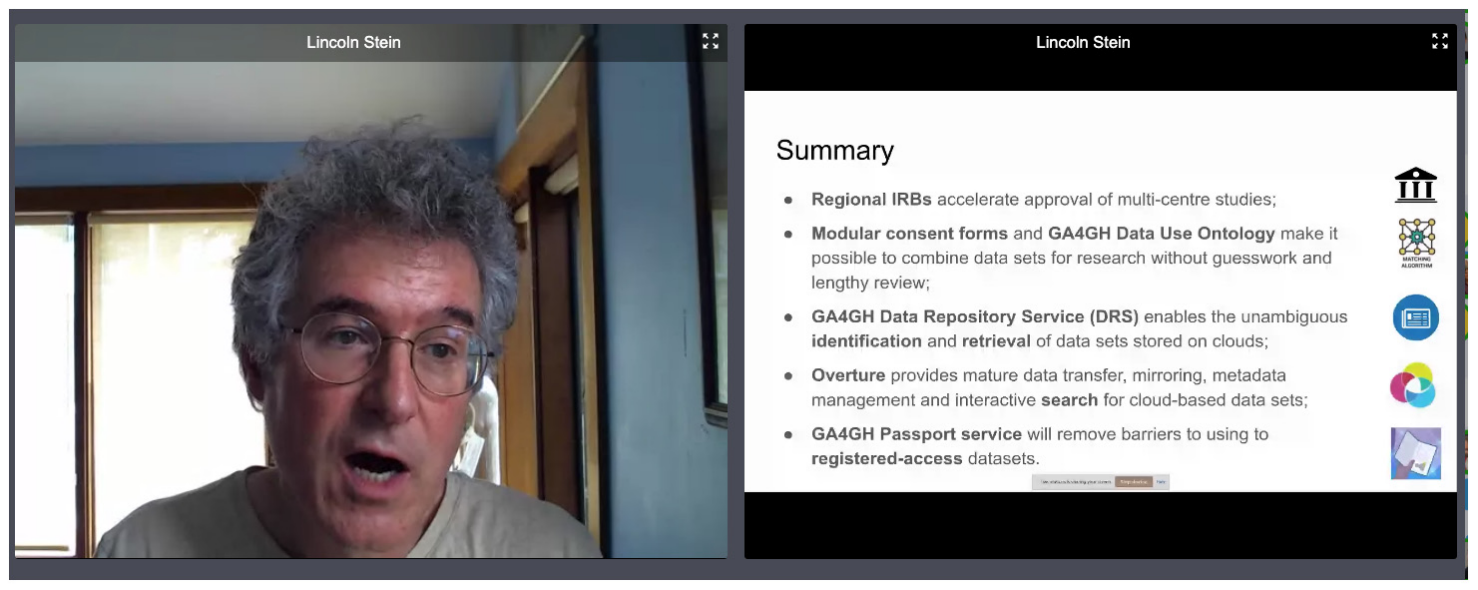
Lincoln Stein speaking at BCC2020.

Lincoln Stein (the head of Adaptive Oncology at the Ontario Institute for Cancer Research) kicked off the meeting in the western hemisphere with the first keynote talk ( Figure 5), 20 years after his keynote at the very first BOSC in 2000. His topic, “Flattening the curve for biomedical data sharing,” was closely aligned with one of BOSC’s pillars, Open Data, as well as being extremely relevant to the current COVID-19 pandemic, which has resulted in a panoply of data related to the disease that is stored in a range of differently structured data stores. Lincoln discussed efforts to collect COVID-related data and make it more widely accessible. His talk is available here; slides are here.

**Figure 6.**
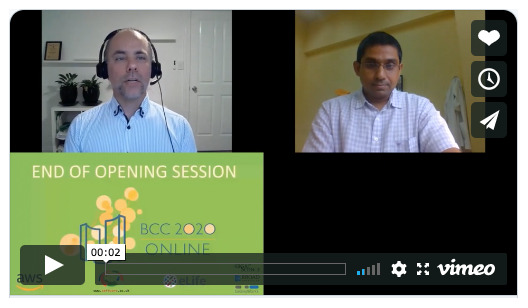
Gareth Price introducing Prashanth Suravajhala. The keynotes were presented live in one hemisphere and recorded in the other.

The first keynote speaker in the East and second in the West was Prashanth Suravajhala (Senior Scientist at Birla Institute of Scientific Research) ( Figure 6). He is a founder of Bioclues.org, India’s largest bioinformatics society, which advocates open access. His topic was “Open minds bring open collaborations.” Prashanth‘s slides are here; a video of his talk is here.

**Figure 7.**
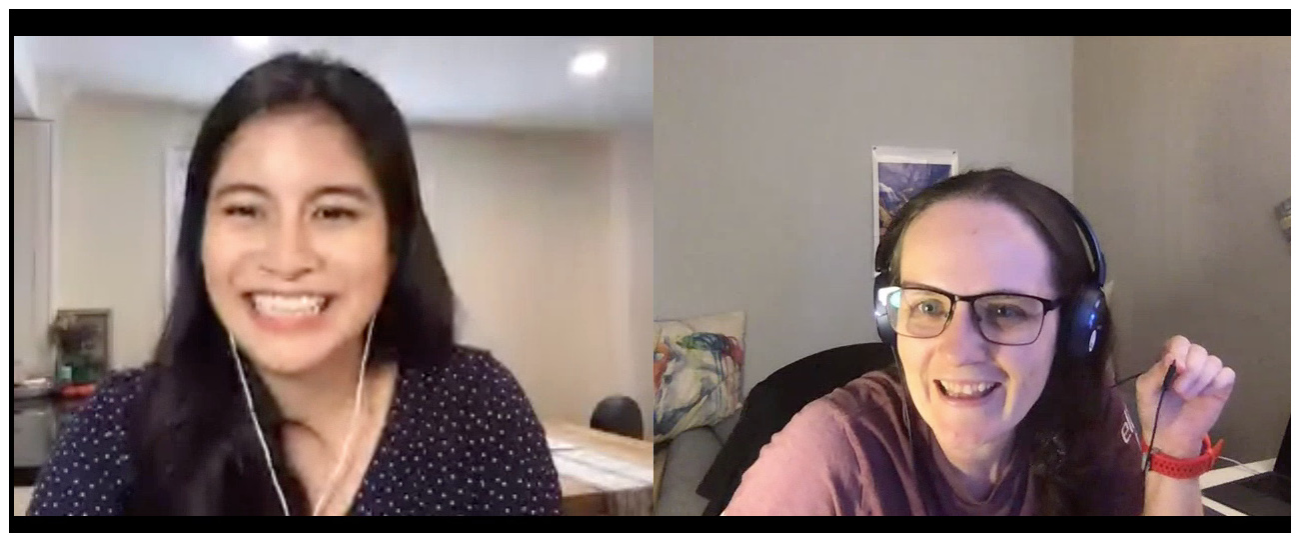
Keynote speaker Abigail Cabunoc Mayes and session chair Yo Yehudi.

Finally, Abigail Cabunoc Mayes (Working Open Practice Lead at Mozilla Foundation) ( Figure 7) closed the conference with a keynote talk entitled “Biased by Default: Exploring Discrimination in Research Code” (her slides are available here, and a video of her talk is here: Part 1, Part 2). Abby observed that the power structure in academia mirrors that of society as a whole. In research, and more specifically in our open source practices, we can begin to shift that power by involving traditionally excluded groups and individuals to share ideas, create together and collaborate in decision-making. Abby used Remo’s table mode to send participants back to discuss the questions, "Where can you include others and share power in your work? Who will you include (who is missing)?" Responses included: opportunities for involving others by delegating tasks, shared development, testing and feedback, teaching, documentation to target members who may need more help, open acknowledgement (without creating unbalanced praise accidentally) and more.

### Talk sessions

In addition to joint keynote talks, BCC2020 had parallel sessions consisting of talks (5-minute lightning talks and 15-minute longer talks) chosen from submitted abstracts. The BOSC 2020 talk schedule can be found here; the full BCC2020 schedule is here. BOSC’s sessions included the usual array of open source, open science and open data topics, as well as our signature “BOSC” session (Building Open Source Communities) devoted to the important aspects that go beyond software and data. The BOSC and Galaxy tracks merged for a special joint session about open science approaches to COVID-19. The complete set of BOSC session topics was:

- Sequencing and analysis
- Open data
- Reproducibility and standards
- Building Open Source Communities (BOSC)
- Developer tools and libraries
- Visualization
- Workflow management systems
- COVID joint session

This year’s conference included a session of Gold Sponsor talks by two of the organizations that sponsored the meeting: AWS and the Software Sustainability Institute. Unlike the regular talks, these were not peer-reviewed.

### Birds of a Feather (BoFs)

Birds of a Feather--informal, self-organized meetups focused on specific topics--have long been a popular part of BOSC. This year’s program included 20 BoFs covering both technical (e.g. Dynamic Visualization of Bioinformatic Data; Computational Biology in the Gloud) and social topics (e.g. Board Games Social; Where is the Bar?). The Open Bioinformatics Foundation (OBF; the parent organization of BOSC) organized a BoF to solicit community feedback on a proposed Code of Conduct that would cover both in-person OBF events and online interaction, and that could be used by OBF member projects as well.

To keep the main conference schedule as short as possible in order to limit screen fatigue, the BoFs were held before and after each 5-hour conference day. Because Remo is not currently designed to support medium-sized gatherings where everyone is able to speak (it is optimized for either 8-person table chats or webinars limited to a few active presenters at a time) some BoFs were held in external platforms such as Zoom. Fortunately, we were able to create “bridges” from the main Remo conference floor to enable attendees to transparently jump to these auxiliary sites.

**Figure 8.**
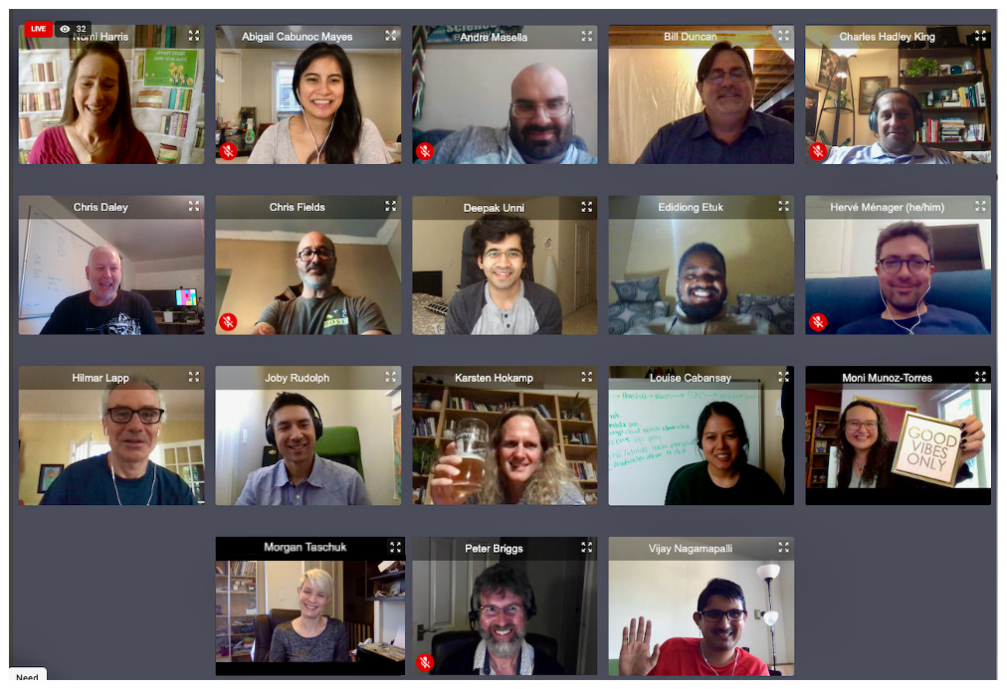
Some of the attendees at the BOSC “party”.

The BOSC and Galaxy online parties, although not officially classified as BoFs, could be considered to fall under that umbrella. Over 40 people attended the BOSC party ( Figure 8), which featured a customized floor plan and “DJed” music. Although it was a pale shadow of the in-person gatherings at previous meetings, the BOSC party was surprisingly enjoyable.

### CoFest

BCC2020 ended with a two-day CollaborationFest (aka CoFest), followed by two additional optional CoFest Encore days for those who just couldn't get enough. This was the 11th annual BOSC-affiliated (and 9th annual Galaxy conference affiliated) collaborative work event. The first was Codefest 2010 in Boston. The event was renamed "CollaborationFest" starting in 2018 to acknowledge the importance of activities besides coding, such as working on documentation, training materials, and use cases.

Over 300 people signed up to participate (for no charge) in CoFest 2020, although the final number of people who participated was estimated at only half that, perhaps due to the difficulty of devoting unstructured time to an online event while regular work and home responsibilities loom, as well as the low bar offered by the free registration.

During CoFest, 25 different project groups formed, using Discord channels for communication. These channels remain open, so collaboration can continue past the end of the meeting.

## Whither BOSC 2021?

At the time this report was written, the plan for BOSC 2021 was not yet settled. It is likely that it will be part of ISMB 2021, whether that meeting is held in person or online. However, if GCC decides to meet online in 2021, it is possible that BOSC will again partner with them. It is hard to predict now whether travel will be feasible in July 2021, but we plan to make a decision by early 2021.

## Consent

All photos in this report are shared under a CC-BY-SA license. All identifiable subjects in the photos were contacted, and they provided written consent to have their photos published in this report.

